# Early Hepatic Oxidative Stress and Mitochondrial Changes Following Western Diet in Middle Aged Rats

**DOI:** 10.3390/nu11112670

**Published:** 2019-11-05

**Authors:** Arianna Mazzoli, Raffaella Crescenzo, Luisa Cigliano, Maria Stefania Spagnuolo, Rosa Cancelliere, Cristina Gatto, Susanna Iossa

**Affiliations:** 1 Department of Biology, Federico II University, Via Cintia,80126 Naples, Italy; arianna.mazzoli@unina.it (A.M.); rcrescen@unina.it (R.C.); luisa.cigliano@unina.it (L.C.); cancelliererosa@gmail.com (R.C.); crigatto51@gmail.com (C.G.); 2 Department of Bio-Agrofood Science, Institute for the Animal Production System in Mediterranean Environment, National Research Council Naples (CNR-ISPAAM), 80147 Naples, Italy; mariastefania.spagnuolo@cnr.it

**Keywords:** middle age, liver mitochondria, oxidative stress, high fat-high fructose diet, inflammation, hepatic cholesterol

## Abstract

To assess the effect of 4 weeks of high fat-high fructose feeding on whole body composition, energy balance, specific markers of oxidative stress and inflammation, and insulin sensitivity in the liver of middle-aged rats, rats (1 year) were fed a diet rich in saturated fatty acids and fructose (HFF rats), mimicking the “Western diet”, and compared with rats of the same age that were fed a low fat diet (LF rats). HFF rats exhibited a significant increase in the gain of body weight, energy, and lipids compared to LF rats. HFF rats also showed hepatic insulin resistance, together with an increase in plasma triglycerides, cholesterol, and tumor necrosis factor alpha. Hepatic lipids, triglycerides and cholesterol were higher in HFF rats, while a significant decrease in Stearoyl-CoA desaturase activity was found in this tissue. A marked increase in the protein amount of complex I, concomitant to a decrease in its contribution to mitochondrial respiration, was found in HFF rats. Lipid peroxidation and Nitro-Tyrosine content, taken as markers of oxidative stress, as well as NADPH oxidase activity, were significantly higher in HFF rats, while the antioxidant enzyme catalase decreased in these rats. Myeloperoxidase activity and lipocalin content increased, while peroxisome proliferator activated receptor gamma decreased in HFF rats. The present results provide evidence that middle-aged rats show susceptibility to a short-term “Western diet”, exhibiting altered redox homeostasis, insulin resistance, and early mitochondrial alterations in the liver. Therefore, this type of dietary habits should be drastically limited to pursue a “healthy aging”.

## 1. Introduction

In the last 20 years, gerontological research has produced significant progresses in the comprehension of the phenomenon of aging, thus opening the prospective that the symptoms and functional consequences of aging can be delayed, reduced, or perhaps eliminated [1]. In particular, it has been shown that the consumption of a high fat-high sugar diet, a common lifestyle factor especially in Western countries, may add to aging to alter cellular function [2,3]. Many studies have been performed on diet-induced impairment of liver function in animal models [4,5]. Although middle-age, an earlier stage of the aging process, is a life phase in which animals are more exposed to develop diet-induced insulin resistance and liver dysfunction [6,7], few data are available on the impact of western diets on physiological responses at middle age [7,8,9], despite the fact that obesity is more frequent in the middle-aged population (40.2%) compared to younger or older adults (32.3% and 37.0%, respectively) [10]. A critical hallmark is the condition of oxidative stress associated to aging and/or overweight and obesity [11]. Among the organs that are more prone to oxidative damage, the liver is deeply involved in the onset of metabolic disorders, including insulin resistance [12], due to its major role in the regulation of systemic lipid and glucose homeostasis [13,14].

Previous findings have shown that the duration of the dietary treatment has a deep impact on the hepatic metabolic impairment that appeared at 4 weeks, plateauing at 8–12 weeks, and then exhibiting a slight amelioration afterwards [15]. In addition, the authors found that both hepatic steatosis and mitochondrial alterations occurred as early as 4 weeks of diet [15], thus suggesting that studies aimed at revealing the interconnection between the metabolic alterations and the intake of obesogenic diets should be focused on this period of dietary treatment. Hence, the aim of the present study was to analyze the short-term impact of a diet rich in saturated fats and sugars on whole body and liver homeostasis in middle aged rats. In particular, the effect of 4 weeks of high fat-high fructose feeding on whole body composition, energy balance, specific markers of oxidative stress and inflammation, as well as insulin sensitivity, was investigated in the liver of middle-aged rats.

## 2. Materials and Methods 

### 2.1. Experimental Design

Male rats of the Sprague-Dawley strain were obtained from Charles River (Calco, Como, Italy). All rats were housed individually at a temperature of 23 °C ± 1 °C with a 12-h light/dark cycle (06.30–18.30). All experimental procedures involving animals met the guidelines set by the Italian Health Ministry and were approved by “Comitato Etico-Scientifico per la Sperimentazione Animale” of the University of Naples “Federico II” (260/2015-PR). 

Middle-aged rats (11 months old) were divided in three groups, each composed of eight rats. One group of rats was euthanized at the beginning of the experiment for determination of initial values of body composition, while the other two groups were fed a high fat-high fructose (HFF) or low-fat diet (LF) for 4 weeks. The composition of the two diets is reported in Table 1. During the dietary treatment, the rats were housed one per cage, and body weight, food and water intake were monitored daily and feces were collected. At the end of the experimental period, the animals were anaesthetized with zolazepam plus tiletamine (1:1) (30 mg kg^−1^ i.p.) and euthanized by decapitation, blood and liver were harvested, and the carcasses used for determination of body composition. 

### 2.2. Metabolic Analyses

Food was withdrawn at 08.00 a.m. After 6 h, basal postabsorptive samples obtained from venous blood from a small tail clip were collected, and then, glucose (2 g kg^−1^) was injected intraperitoneally. Blood samples were collected after 20, 40, 60, 90, 120, and 150 min and placed in EDTA coated tubes. The blood samples were centrifuged at 1400 g for 8 min at 4 °C and plasma was isolated. Plasma glucose concentration was measured by a colorimetric enzymatic method (Pokler Italia, Pontecagnano, Italy). Plasma insulin concentration was measured using an ELISA kit (Mercodia AB, Uppsala, Sweden) in a single assay to avoid interassay variations. Hepatic insulin resistance index was calculated according to Abdul-Ghani et al. [16]. Briefly, since the increase in plasma glucose and insulin concentrations in the period 0–30 minutes after the glucose injection is proportional to the degree of hepatic insulin resistance [16], we calculated the rise in plasma glucose and insulin concentrations during the first 30 minutes of the glucose load by measuring the area under the curve (AUC) of plasma glucose and insulin. Then, hepatic insulin resistance index was calculated as: (glucose AUC 0–30) × (insulin AUC 0–30) [16].

The same procedure was used to isolate plasma from systemic blood collected at the moment of euthanasia. Commercial kits adopting colorimetric enzymatic methods were used to measure the plasma concentrations of total cholesterol, low density lipoprotein (LDL) cholesterol, high density lipoprotein (HDL) cholesterol, triglycerides, and alanine aminotransferase (ALT) (SGM Italia, Rome, Italy). Plasma tumor necrosis factor alpha (TNF-α) concentrations were determined using a rat-specific ELISA assay (R&D Systems, MN, USA).

Body energy, lipid, and protein content were measured as previously described [17]. Briefly, the alimentary tract was cleaned of undigested food and the carcasses were then autoclaved. After dilution in distilled water and subsequent homogenization of the carcasses, duplicate samples of the homogenized carcass were analyzed for energy content by a bomb calorimeter. Total body lipids, as well as hepatic lipids, were measured by the Folch extraction method [18]. The energy as lipid, calculated from body lipids by using the coefficient of 39.2 kJ g^−1^, was then subtracted from total body energy to obtain the energy as protein. Liver triglycerides and cholesterol were measured as described above for plasma. Liver ceramide content was evaluated by enzyme-linked immunosorbent assay (ELISA) as reported previously [19]. Briefly, ELISA microplates were coated overnight at 4 °C with hepatic lipids extracted with the Folch method [18] (70 μL in methanol). Plates were then blocked with 10 mM phosphate buffer, 140 mM NaCl, 0.1% Tween, pH 7.4 supplemented with 1% bovine serum albumin for 1 h at 37 °C. After washing, the plates were incubated with monoclonal anti-ceramide antibody (Sigma, MO, USA, 2 μg/mL) for 1 h at 37 °C, washed and incubated with peroxidase-conjugated goat anti-mouse IgM (Sigma, MO, USA, 1:5000 dilution) for 1 h at 37 °C. After washings, 100 μL of a color development solution (20 mg of o-Phenylenediamine dihydrochloride in 50 mL of 70 mM Na_2_HPO_4_, 30 mM citric acid, pH 5, supplemented with 120 μL of 3% H_2_O_2_) were added to the wells. After 15 min at 37 °C, the reaction was stopped by the addition of 50 μl of 2.5 M H_2_SO_4_ and the absorbance was measured at 492 nm. All tests were done in triplicate. Immunoreactivity was normalized to starting tissue weight. Negative control reactions included omission of primary antibody.

Energy balance measurements were conducted by the comparative carcass technique over the experimental period. Briefly, daily food consumption was monitored by weighing daily the food left in the cage and subtracting it from the food placed in the cage at the same time of the previous day. Energy content of the diet was assessed by a bomb calorimeter and gross energy intake was then calculated. Energy content in feces and urine was measured by a bomb calorimeter and the values were subtracted from gross energy intake to obtain metabolizable energy (ME) intake. Body energy, lipid, and protein gain were calculated as the difference between the final and initial content of body energy, fat, and protein. Energy expenditure was determined as the difference between energy gain and ME intake, and the energetic efficiency was calculated as the percentage of total energy gain per ME intake.

### 2.3. Hepatic Mitochondrial Function 

Liver was homogenized (1:1000, *w/v*) in Mir05 medium containing 110 mM sucrose, 60 mM K-lactobionate, 20 mM Hepes, 20 mM taurine, 10 mM KH_2_PO_4_, 6 mM MgCl_2_, 0.5 mM EGTA, 0.1% fatty acid free BSA, pH 7.0. 

Samples (2 mg of tissue in 2 mL of of Mir05 medium) were incubated into calibrated Oxygraph-2 k (O_2_k, Oroboros Instruments, Innsbruck, Austria) 2 ml-chambers at 37 °C ± 0.001 °C and oxygen concentration (μM) and oxygen flux per tissue mass (pmol O_2_ s−1•mg−1) was recorded real-time using DatLab software (OROBOROS INSTRUMENTS, Innsbruck, Austria). 

After addition of the homogenate samples, a Substrate, Uncoupler, Inhibitor Titration (SUIT) protocol was applied to assess mitochondrial changes. Leak respiration through complex I of the respiratory chain (CI) was evaluated by adding the substrates malate (0.5 mM), pyruvate (5 mM), and glutamate (10 mM). ADP 2.5 mM was then added, to asses phosphorylating respiration with electron transfer supported by Complex I (CI_P_). Maximal phosphorylating respiration with electron input from Complex I and II (CI&II_P_) was achieved by adding succinate at 10mM. Oligomycin at 2.5 mM was added to inhibit ATP synthase, followed by the addition of the uncoupler carbonylcyanide p-trifluoromethoxyphenyl-hydrazon (FCCP, 0.5 mM), to evaluate maximum capacity of the electron transport chain (CI&II_ETS_). Rotenone (0.5 µM) was added to inhibit CI and determine the maximal capacity supported by CII alone (CII_ETS_). Residual oxygen consumption was established by the addition of the inhibitor Antimycin A (2.5 mM) and the resulting value was subtracted from the fluxes in each run to correct for non-mitochondrial respiration. All samples were run in duplicates and the mean was used for analysis. Oxygen consumption rates were then used to calculate flux control factor (FCF) for complex I- or complex II-linked substrates as follows: FCF_CI_ = 1- (CII_ETS_/CI + II_ETS_); FCF_CII_ = 1-(CI_p_/CI + II_P_), according to Burtscher et al. [20].

The stimulating effect of 10 mM exogenous cytochrome c on mitochondrial respiration in the presence of complex I-linked substrates and ADP was tested to evaluate mitochondrial integrity.

### 2.4. Hepatic Oxidative Status and Inflammation

Lipid peroxidation was determined by measuring thiobarbituric acid reactive substances (TBARS) [21]. Liver homogenates were prepared in KCl 175 mM, Tris 10 mm, pH 7.5 (1:8 *w/v*) and processed as described previously [22]. 

Catalase activity was measured in liver homogenates prepared in 50 mM phosphate buffer, pH 7.0 (1.50 *w/v*), by monitoring the decrease in absorbance at 240 nm due to the decomposition of H_2_O_2_ [23]. 

Superoxide dismutase activity (SOD) was measured in liver homogenates prepared in 50 mM phosphate buffer, pH 7.0 (1.50 *w/v*) by the method of Flohè & Otting [24], as detailed previously [19]. 

NADPH oxidase activity in liver membrane fractions was assayed according to a modification of the method of Bettaieb et al. [25]. Briefly, liver tissue (1:10 *w/v*) was homogenized in ice-cold Krebs buffer and then centrifuged at 800 g, at 4 °C for 10 min. The supernatant was collected and then centrifuged at 30,000 *g* for 2 h at 4 °C. The pellet (membrane fraction) was resuspended in Krebs buffer and protein concentration measured. Aliquots containing 100 μg of protein were added to Krebs buffer containing NADPH (500 μM). The change in absorbance at 340 nm was followed for 10 min at 30 s intervals. Enzyme activity was expressed as nmol/min x mg protein. 

Nitro-Tyrosine (N-Tyr) titration in liver homogenates was carried out by ELISA essentially as previously reported [22]. 

Myeloperoxidase (MPO) activity was assessed as reported by Kim et al. [26] and described in detail previously [22] on liver samples (100 mg) that were homogenized in 1 mL of hexadecyltrimethylammonium bromide (HTAB) buffer (0.5% HTAB in 50 mM phosphate buffer, pH 6.0). 

### 2.5. Lipogenesis in the Liver

Stearoyl CoA desaturase (SCD) activity was measured polarographically in liver homogenates prepared in KCl 175 mM, Tris 10 mm, pH 7.5 (1:8 *w/v*) at 37 °C in a solution containing 0.1 M K_2_HPO_4_, pH 7.4, 1 μM myxothiazol, 0.12 mM NADH and 0.06 mM stearoyl CoA as cyanide (5 mM)-sensitive [27], and myxothiazol-insensitive oxygen consumption. 

Fatty acid synthase (FAS) activity was measured in liver homogenates prepared in KCl 175 mM, Tris 10 mm, pH 7.5 (1:8 *w/v*) according to Penicaud et al. [28]. Briefly, samples were incubated in a buffer containing KH_2_PO_4_ 0.1 M, pH 6.5, acetyl-CoA 60 μM, malonil-CoA 90 μM, and NADPH 300 μM. The change in absorbance at 340 nm was measured, and one unit of FAS activity was defined as that degrading 1 μmoL of NADPH per minute at 37 °C.

### 2.6. Western Blotting of Hepatic Proteins

Proteins were extracted from livers by diluting tissue samples 1:1 with lysis buffer (20.0 mmoL/L Tris, pH 8, 5% glycerol, 138 mM NaCl, 2.7 mM KCl, 1% NP-40, 5 mM EDTA, 5% protease inhibitor cocktail, 1% phosphatase inhibitor cocktail). Homogenates were centrifuged at 15,000 g for 15 min at 4 °C, and the supernatants were then collected and used for the quantification of several proteins, namely Apolipoprotein E (ApoE) p-Akt, p-Erk, lipocalin, LDL receptor, peroxisome proliferator activated receptor alpha (PPARα), peroxisome proliferator activated receptor gamma (PPARγ), and mitochondrial respiratory complexes I to V. 

Aliquots (40 µg for the detection of ApoE and respiratory complexes or 20 µg for the other proteins) were denaturated in Laemmli’s buffer (60 mM Tris pH 6.8, 10% sucrose, 2% SDS, 4% β-mercaptoethanol, 0.02% bromophenol blue) and loaded on a 12.5% SDS–polyacrylamide gel. After the run, the gels were transferred on polyvinylidene difluoride membranes (Millipore, Billerica, MA, USA) at 0.8 mA/cm^2^ for 90 min. The membranes were preblocked in PBS, 3% bovine albumin serum, 0,3% Tween 20 (for p-Akt, p-Erk, lipocalin, LDL receptor, PPARα, and PPARγ) or with T-TBS containing 5% non-fat milk (for ApoE and respiratory complexes) for 1 h and then incubated overnight at 4 °C with antibodies for p-Akt (Cell Signaling, Danvers, MA, USA; diluted 1:1000 in blocking buffer), p-Erk (Cell Signaling, Danvers, MA, USA; diluted 1:1000 in blocking buffer), lipocalin (Thermo Fisher, Rock Ford, USA; diluted 1:200 in blocking buffer), LDL Receptor (Abcam, Cambridge, UK, 1:200 in blocking buffer), PPAR-α (Thermo Fisher, IL, USA; 0,5 mg/mL in blocking buffer), PPAR-γ (Thermo Fisher, IL, USA; 1:1000 in blocking buffer), ApoE (Millipore, Billerica, MA, USA; diluted 1:500 in T-TBS containing 3% BSA), or anti-Oxphos (Abcam, Cambridge, UK; 1:400 dilution in T-TBS containing 3% BSA). Membranes were washed and then incubated for 1 h at room temperature with HRP-conjugated secondary antibodies (Promega, Madison, WI, USA, diluted 1:5000 for p-Akt, p-Erk, lipocalin, LDL receptor, PPAR-α and PPAR-γ; or Sigma-Aldrich, St Louis, MO, USA, diluted 1:500,000 for ApoE or 1:60,000 for respiratory complexes). For p-Akt, p-Erk, lipocalin, LDL receptor, PPAR-α, and PPAR-γ, the membranes were washed and incubated at room temperature with a chemiluminescent substrate, Immobilon (Millipore Corporation, Billerica, MA 01821, USA). For loading control, Akt was detected with polyclonal antibody (Cell Signaling, Danvers, MA, USA; diluted 1:1000 in blocking buffer) and used to normalize the p-Akt signal, total Erk was detected with monoclonal antibody (Cell Signaling, Danvers, MA, USA; diluted 1:1000 in blocking buffer) and used to normalize p-Erk, while actin was detected with polyclonal antibody (Sigma-Aldrich, St Louis, MO, USA; diluted 1:1000 in blocking buffer) and used to normalize the lipocalin, LDL Receptor, PPAR-α and PPAR-γ signals. Quantitative densitometry of the bands was carried out by analyzing chemidoc images using Image Lab Software (Biorad Laboratories S.r.l., Segrate (MI)–Italy).

For ApoE and respiratory complexes, the membranes were washed and incubated with the Excellent Chemiluminescent detection Kit (ElabScience, Microtech, Naples, Italy). For loading control, actin was detected with monoclonal antibody (Sigma-Aldrich; 1000 dilutions in T-TBS containing 0.25% non-fat milk) and used to normalize ApoE and respiratory complexes signal. Quantitative densitometry of the bands was carried out by analyzing digital images of X-ray films exposed to immunostained membranes by using Un-Scan-It gel software (Silk Scientific, UT, USA).

### 2.7. Statistical Analysis

Data are reported as mean values ± SEM. GraphPad Prism 8 (GraphPad Software, San Diego, CA) was used for statistical analysis by applying two-tailed, unpaired Student’s t-test. *p* < 0.05 was considered significant. 

## 3. Results

### 3.1. Body and Liver Composition and Plasma Parameters

The HFF rats exhibited a significant increase in body weight gain, body energy gain, and body lipid gain. On the other hand, body protein gain (calculated as the difference between body energy gain and lipid energy gain and expressed in KJ) was significantly lower in HFF rats compared to controls (Table 2).

**Figure 1:**
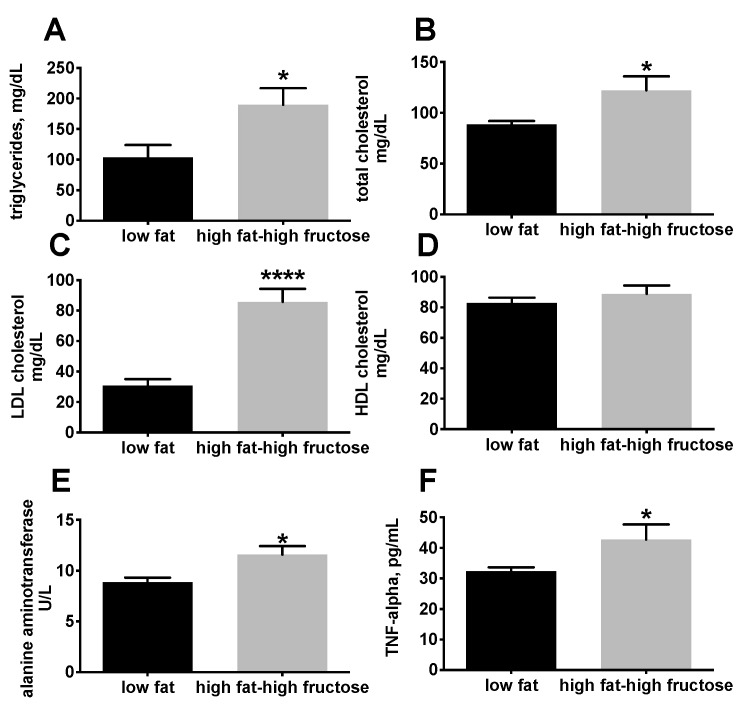
Plasma levels of markers of lipid homeostasis, inflammation, and hepatic necrosis in middle aged rats fed a low fat or high fat-high fructose diet for 4 weeks. Plasma lipid profile was assessed by determining triglycerides (**A**), total cholesterol (**B**), LDL cholesterol (**C**), and HDL cholesterol (**D**). Plasma levels of alanine aminotrasferase were assessed as marker of liver necrosis (**E**), while plasma TNF-α was measured as marker of inflammation (**F**). Values are the means ± SEM of eight rats. * *p* < 0.05, **** *p* < 0.0001 compared to low fat diet (two-tailed, unpaired, student’s t-test).

Plasma lipid profile shows a significant increase in triglycerides (Figure 1A), total cholesterol (Figure 1B), and LDL cholesterol (Figure 1C) in HFF rats compared to LF. In addition, plasma levels of ALT, a reliable index of hepatocellular necrosis [29], were significantly higher in HFF rats compared with LF rats (Figure 1E). Plasma concentrations of TNF-α were assessed as marker of systemic inflammation, and the results show that TNF-α was significantly increased in HFF rats compared to LF after 4 weeks of dietary treatment (Figure 1F). 

**Figure 2:**
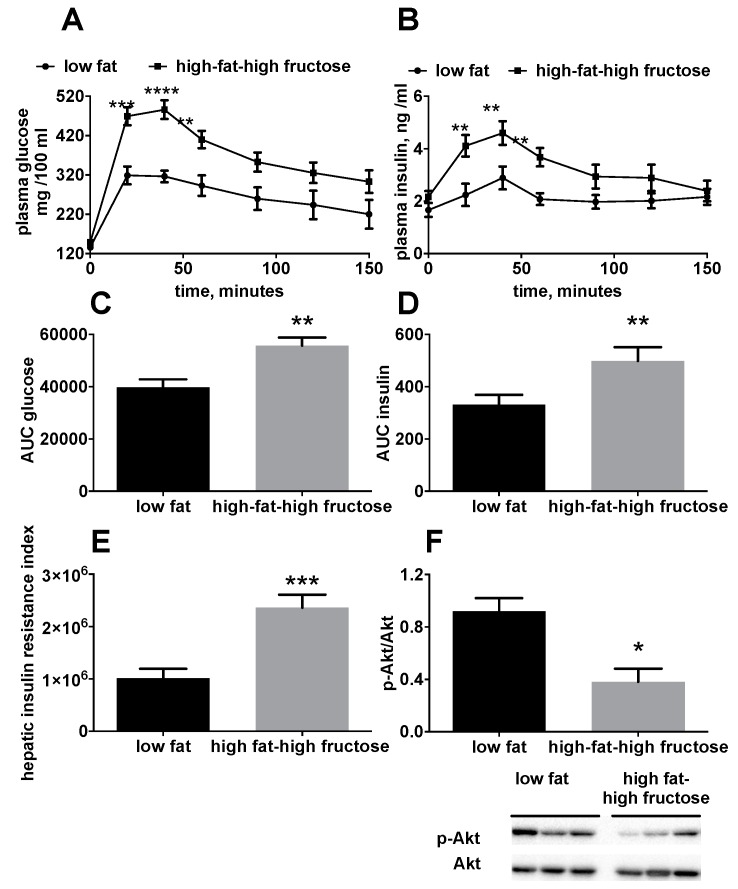
Glucose homeostasis in middle aged rats fed a low fat or high fat-high fructose diet for 4 weeks. The day before the sacrifice, rats were fasted for six hours, basal postabsorptive plasma samples were collected, glucose (2 g/kg b.w.) was injected intraperitoneally and further plasma samples were collected after 30, 60, 90, 120, and 150 minutes. Plasma glucose (**A**) and insulin (**B**) levels during the glucose tolerance test; area under the curve of plasma glucose (**C**) and insulin (**D**); hepatic insulin resistance index calculated during the first 30 minutes of the glucose tolerance test (**E**) and hepatic content of the active, phosphorylated form of the kinase Akt (with representative blot images) (**F**), assessed through western blot and normalized to Akt. Values are the means ± SEM of eight rats. * *p* < 0.05, ** *p* < 0.01, *** *p* < 0.001, **** *p* < 0.0001 compared to low fat diet (two-tailed, unpaired, student’s t-test).

In order to have a general picture of the glucose balance, plasma glucose and insulin levels, as well as the activation of Akt, were investigated. Significantly higher plasma glucose (Figure 2A,C) and insulin levels (Figure 2B,D) were found in HFF rats compared to the LF, during the glucose load. In addition, hepatic insulin resistance index, calculated during the early phase of the glucose tolerance test, was found to be significantly higher in HFF rats compared to LF rats (Figure 2E). Hepatic insulin sensitivity was also assessed by determining the degree of phosphorylation of the kinase Akt, a downstream effector of insulin signaling, which was found to be significantly lower in HFF rats compared to LF rats (Figure 2F).

**Figure 3:**
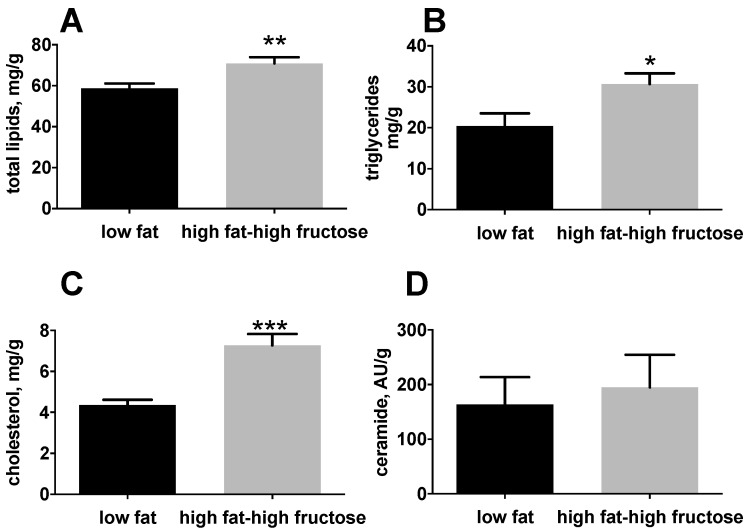
Hepatic lipid composition in middle aged rats fed a low fat or high fat-high fructose diet for 4 weeks. Lipid composition in the liver was assessed by measuring total lipids (**A**), triglycerides (**B**), total cholesterol (**C**), and ceramides (**D**). Values are the means ± SEM of eight rats. * *p* < 0.05, ** *p* < 0.01, *** *p* < 0.001 compared to low fat diet (two-tailed, unpaired, student’s t-test).

With the aim to clarify the effect of the HFF diet on the liver, we performed an analysis of lipid composition of this important metabolic organ. HFF rats exhibited a significant increase in hepatic lipids (Figure 3A), triglycerides (Figure 3B), and cholesterol (Figure 3C) compared to the LF rats, while no differences were found in ceramides levels (Figure 3D).

**Figure 4:**
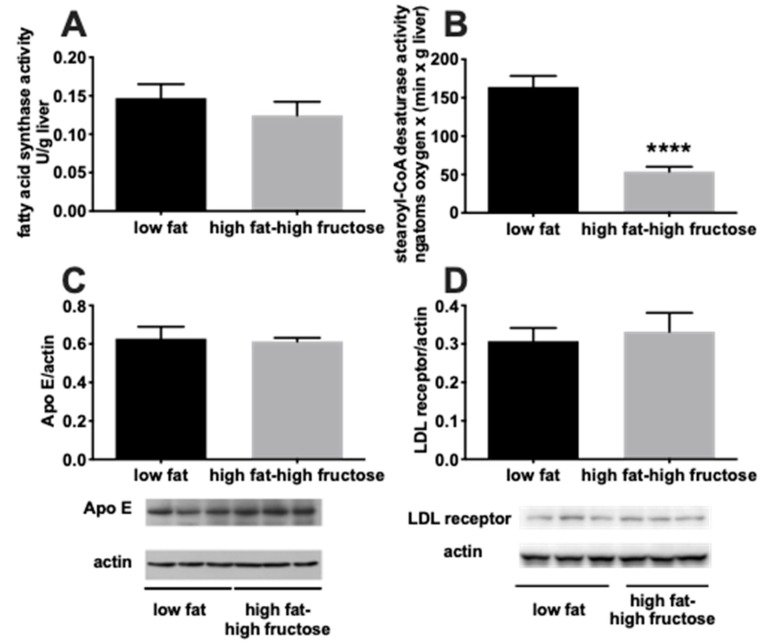
Hepatic markers of de novo lipogenesis and cholesterol handling in middle aged rats fed a low fat or high fat-high fructose diet for 4 weeks. The enzymatic activity of fatty acid synthase (**A**) and stearoyl-CoA-desaturase (**B**) was assessed to evaluate hepatic de novo lipogenesis, while the protein content of ApoE (with representative blot images) (**C**) and LDL receptor (with representative blot images) (**D**) were taken as markers of cholesterol handling. Values are the means ± SEM of eight rats. **** *p* < 0.0001 compared to low fat diet (two-tailed, unpaired, student’s t-test).

To gain further insight into the possible mechanisms underlying the altered hepatic lipid balance, we evaluated the activity of two major enzymes involved in the pathway of de novo lipogenesis, namely FAS and SCD, together with the hepatic protein content of ApoE and LDL receptor (Figure 4). A significant decrease in SCD activity was found in HFF rats compared to the LF rats (Figure 4B), while no difference was found in FAS activity (Figure 4A), nor in the hepatic protein content of ApoE (Figure 4C) and LDL receptor (Figure 4D).

### 3.2. Liver Oxidative Status and Inflammation Markers

**Figure 5:**
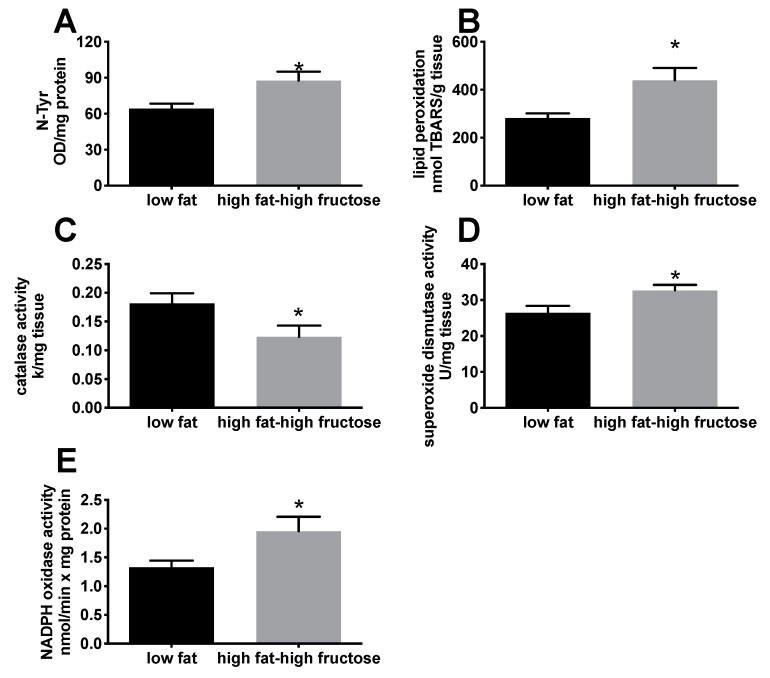
Oxidative status in the liver from middle aged rats fed a low fat or high fat-high fructose diet for 4 weeks. Oxidative damage to proteins (**A**) and lipids (**B**), together with antioxidant enzymes catalase (**C**) and superoxide dismutase (**D**) and NADPH oxidase activity (E). Values are the means ± SEM of eight rats. * *p* < 0.05 compared to low fat diet (two-tailed, unpaired, student’s t-test).

N-Tyr content and lipid peroxidation were analyzed as markers of oxidative damage to proteins and lipids, respectively. Both markers were significantly higher in HFF rats compared with LF rats, showing an increase of the oxidative damage to proteins (Figure 5A) and lipids (Figure 5B) in HFF rats. This result was also confirmed by the analysis of NADPH oxidase activity, one of the major sources of reactive oxygen species (ROS), which was significantly higher in HFF rats compared to the LF (Figure 5E). In addition, the antioxidant enzyme catalase significantly decreased (Figure 5C), while SOD significantly increased (Figure 5D), in HFF rats.

**Figure 6:**
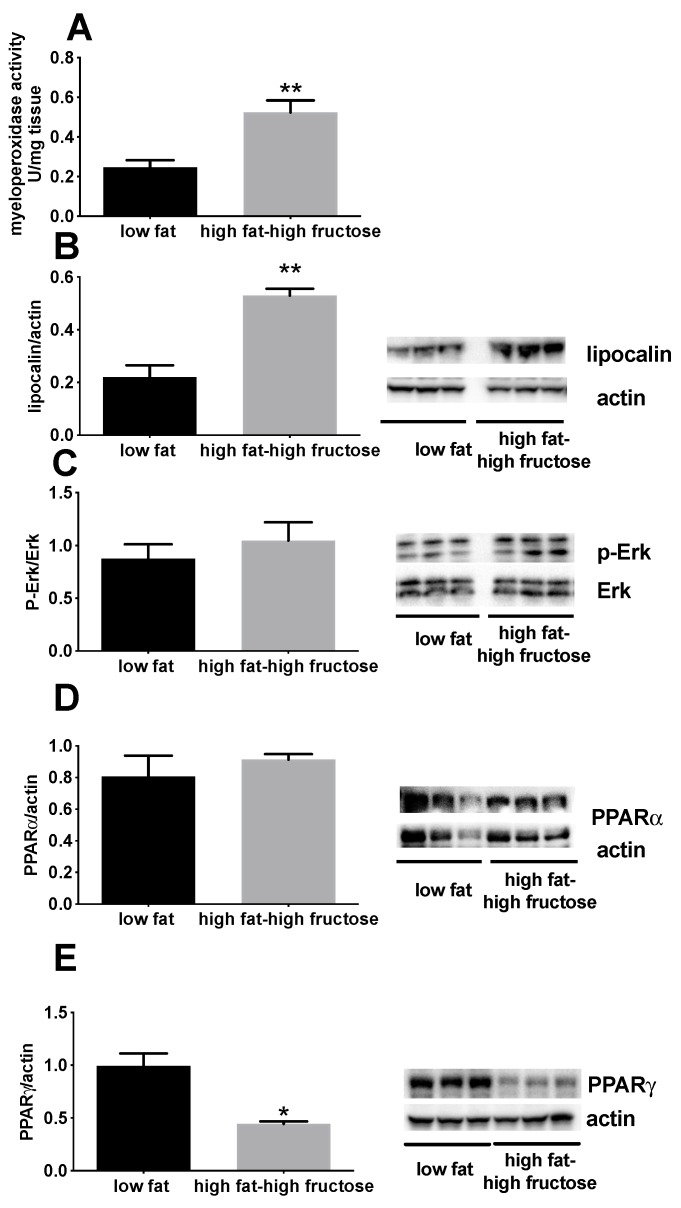
Markers of inflammation in the liver of middle aged rats fed a low fat or high fat-high fructose diet for 4 weeks. Inflammatory status of the liver was assessed by measuring the activity of myeloperoxidase (**A**), together with the protein content of lipocalin (with representative blot images) (**B**), p-Erk (with representative blot images) (**C**), PPARα (with representative blot images) (**D**) and PPARγ (with representative blot images) (**E**). Values are the means ± SEM of eight rats. * *p* < 0.05, ** *p* < 0.01 compared to low fat diet (two-tailed, unpaired, student’s t-test). PPAR = peroxisome proliferator activated receptor.

**Figure 7:**
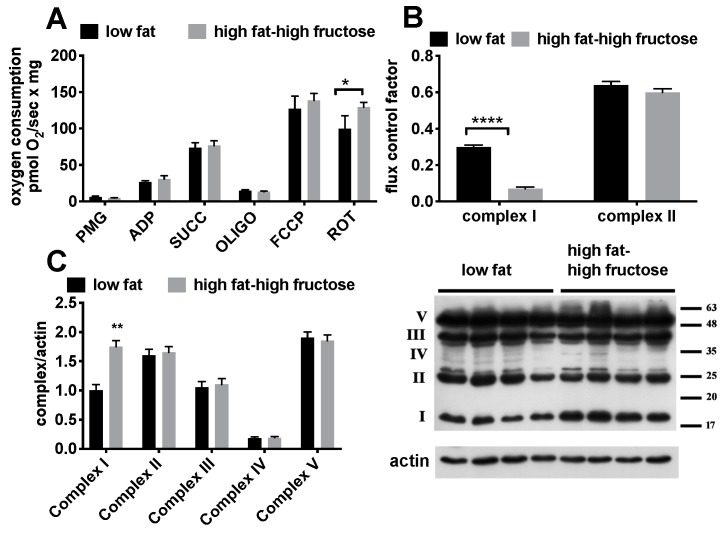
Respiratory rates (**A**), flux control factors (**B**), and protein content of the respiratory complexes (with representative blot images) (**C**) in the liver from middle aged rats fed a low fat or high fat-high fructose diet for 4 weeks. Values are the means ± SEM of eight rats. * *p* < 0.05, ** *p* < 0.01, **** *p* < 0.0001 compared to low fat diet (two-tailed, unpaired, student’s t-test).

MPO activity and lipocalin can be used as surrogate markers of inflammation. In fact, it has been shown that the activity of MPO, solubilized from the inflamed tissue, is directly proportional to the number of neutrophils seen in histological sections [30], and lipocalin plays a key role in modulating the acute-phase response [31]. MPO activity (Figure 6A) and lipocalin content (Figure 6B) were found to be significantly higher in HFF rats (Figure 7). In addition, a significant decrease in the amount of PPARγ, a transcription factor with known anti-inflammatory activity, was found in the liver of HFF rats (Figure 6E), while no variation was found in p-Erk (Figure 6C) and PPARα (Figure 6D).

### 3.3. Intrinsic Mitochondrial Function

Since the condition of oxidative stress found in the liver might be strictly linked to mitochondrial dysfunction and/or alteration in the level of respiratory complexes, we then focused on the analysis of these organelles (Figure 7). As for mitochondrial oxygen consumption rates, a significant diet-induced increase in FCCP-stimulated respiration with complex II-linked substrate succinate was found in HFF rats, while all the other values were not significantly affected (Figure 7A). In addition, a significant decrease in FCF for complex I -linked respiration was found in HFF rats (Figure 7B). Parallel determination of the amount of the various respiratory complexes revealed a marked increase in the amount of complex I, while no variation was observed for the other complexes (Figure 7C).

## 4. Discussion

Middle age is an earlier stage of the aging process, during which gradual physical changes and some chronic illness may occur, thus affecting the outcomes at older ages. Many studies have emphasized the impact of diets on animal models of aging. However, few data are available on the impact of dietary fats and sugars on physiological responses at middle age. In our effort to trace the molecular events that promote the development of an imbalance in hepatic metabolism, we aimed to delineate diet-related effects at an early stage of disease development. Therefore, we have chosen a 4-week period to carry out dietary intervention in 11 months-old rats.

The rat model used in this study highly resembles dietary habits of Western countries, i.e., consumption of a high-saturated fat diet combined with a high fructose intake. The increased plasma levels of triglycerides and LDL cholesterol confirm that the above diet was able to induce metabolic alterations similar to those found in humans [32].

HFF rats exhibited a 60% increase in body fat mass, suggestive of obesity onset subsequent to increased fat and sugar intake. The protein gain was negative in control rats, so that final body protein content was significantly lower compared to initial values, indicating that rats are losing protein mass, a condition typical of increasing age [33]. In addition, the protein gain in HFF rats was significantly lower compared to control rats (indicating that the loss of protein mass was exacerbated by HFF diet). This loss was reflected in lower body protein content in HFF rats compared to LF rats, although the values did not reach statistical significance. We have previously found that adult (90 days) rats fed an HFF diet showed increased body lipid gain, while protein gain was not affected by diet [34,35]. Therefore, it appears that middle-aged rats are metabolically more vulnerable to the effects of HFF diet, thus pointing to dietary habits of middle-aged population as a critical factor in the maintenance of body lean mass and hence of a well-being state.

In addition to the imbalance in lipid metabolism, the HFF diet determined insulin resistance. The glucose tolerance test was impaired, and the hepatic insulin resistance index was increased in HFF rats. In line with this result, at the molecular level, the amount of the phosphorylated, active form of kinase Akt, a downstream effector of insulin signaling, was lower in HFF rats.

Another metabolic perturbation found in HFF rats was the altered lipid profile, with increases in triglycerides and total and LDL cholesterol. In addition, increased markers of systemic inflammation (TNF-α) and liver necrosis (plasma ALT) were evident in these rats. Since the liver plays a central role in lipid handling and is the major site of fructose metabolism, we evaluated the metabolic effects of HFF diet on this organ. In agreement with previous findings obtained in adult rats [34], we found that HFF diet elicited an increased triglyceride deposition in the liver, coupled with enhanced hepatic cholesterol content. The increased hepatic triglyceride content could arise from the lack of inhibition of de novo lipogenesis in the liver, which usually takes place following the intake of high fat diets, to counteract increased lipid flow coming from the diet [36]. This metabolic response is in line with our previous findings obtained in the liver of adult rats after 2 weeks of HFF diet [34]. Similarly, the marked downregulation of SCD activity is in agreement with our previous findings [34] and with those showing that a diet-induced fatty liver is associated with the downregulation of hepatic Scd transcript and de-dimerization of the synthesized protein in a rat model [37]. Interestingly, low hepatic SCD activity is associated with fatty liver and insulin resistance in obese humans [38].

To shed light on the possible mechanism leading to the increased hepatic cholesterol content in HFF rats, we looked at ApoE and LDL receptor protein content in the liver, but no significant changes were found, thus ruling out the possibility that these two proteins, which play a significant role in lipoprotein secretion and uptake [39], are involved in the process. We can speculate that the downregulation of SCD activity found in HFF rats could play a role in the increased hepatic cholesterol. In fact, SCD synthesize monounsaturated fatty acids, mainly palmitoleic and oleic fatty acids, which are the preferred fatty acids utilized for the formation of cholesterol esters and their inclusion into the very low density lipoproteins [40]. Thus, it is possible that the excess cholesterol coming to the liver from the diet is only partially exported, while the major fraction remains in the liver.

HFF diet was associated with increased hepatic oxidative stress to lipids and proteins. Oxidative stress arises when either increased ROS production or decreased antioxidant defense occurs. One factor potentially contributing to increased oxidative stress is the significant decrease in the antioxidant enzyme catalase. Among the cellular sites involved in the production of ROS, mitochondria play a prominent role. Therefore we investigated the respiratory function of hepatic mitochondria and we found a diet-induced impairment in complex I-driven respiration, associated with a compensatory increase in the amount of complex I. It should be noted that we have previously found a significant decrease in complex I-linked respiration in young (30 days old) rats after 30 days of a lipid-rich diet [41]. Thus, it is possible that, independent of the age of the animals, oxidative damage specifically to mitochondrial complex I takes place in the liver following an increase in the intake of dietary lipids. In agreement with this suggestion, it has been found that the activity of hepatic mitochondrial complex I is specifically downregulated following high fat feeding [42] and that this complex is more sensitive to oxidative damage compared to the others [43].

Another important cellular source of ROS production is the enzyme NADPH oxidase. The significant increase of this enzyme found in HFF rats is indicative of increased ROS production through this pathway. In fact, NADPH oxidase is a multiprotein complex found in all types of liver cells, including hepatocytes [44], which may cause oxidative stress by reducing molecular oxygen to superoxide and hydrogen peroxide. A role for NADPH oxidase in the pathogenesis of liver diseases has been suggested, on the basis of the findings that NADPH oxidase-deficient mice are resistant to liver fibrosis [45]. In addition, it has been shown that both the gene expression of the molecular components of NADPH oxidase and its enzyme activity are increased in the liver of mice fed a lipid-rich diet [46] and ROS production through this pathway has been found enhanced in rats fed a high fat-high fructose diet [47]. It should be emphasized that a number of factors may induce NADPH oxidase activity, including saturated fatty acids [48] and TNF-α [49]. 

The increased oxidative stress in the livers of HFF rats is accompanied by increased markers of inflammation, such as myeloperoxidase activity and lipocalin content. Of note is the finding that PPARγ protein content is markedly downregulated in HFF rats, thus probably contributing to tissue inflammation, considering the anti-inflammatory role of this transcription factor [50]. The evidenced increase in hepatic lipocalin is interesting, since this protein, whose main source is the liver [51], is currently regarded both as a marker of obesity [52] and considered to exert a key role in the modulation of the acute phase response [53]. A marked upregulation of lipocalin in the liver has been found in parallel with altered mitochondrial function and increased inflammation after 4 weeks of high fructose diet [54]; thus, it can be suggested that the increase in hepatic lipocalin found here is dependent on the high fructose content of the HFF diet.

## 5. Conclusions

In conclusion, the present results provide evidence that middle-aged rats show susceptibility to a short-term “Western diet”, exhibiting altered redox homeostasis, insulin resistance and early mitochondrial alterations in the liver. Future studies addressing the response to high-fat/high-fructose diet of younger rats in comparison with middle-aged ones will allow to keep further insight into the interaction between age and diet. In the context of the midlife, our data point out that this type of dietary habits should be drastically limited or avoided to pursue a “healthy aging”.

## Figures and Tables

**Table 1:** Diet composition.

	Low Fat	High Fat-High Fructose
**Component, g/1000 g**		
Standard Chow ^a^	395.3	231.5
Sunflower oil	19.3	19.3
Casein	59.7	133.3
Water	175.7	175.4
AIN-93 Mineral mix	11.4	11.4
AIN-93 Vitamin mix	3.2	3.2
Choline	0.7	0.7
Methionine	0.9	0.9
Cornstarch	333.8	-------
Butter	-------	129.8
Fructose	-------	294.6
**Energy content and composition**		
ME content, kJ/g ^b^	11.2	14.9
Lipids, J/100 J	10.5	39.3
Proteins, J/100J	19.9	19.8
Complex carbohydrates, J/100 J	63.9	7.5
Simple sugars, J/100 J	5.7	33.4

^a^ 4RF21, Mucedola, Italy; ^b^ Estimated by computation using values (kJ/g) for energy content as follows: Protein 16.736, lipid 37.656, and carbohydrate 16.736. ME = metabolizable energy, AIN= American Institute of Nutrition.

**Table 2:** Body composition and energy balance.

	Initial Values	Low Fat	High Fat-High Fructose
**Body Composition**			
Initial body weight, g		642.0 ± 40.0	643.0 ± 40.0
Final body weight, g		654.0 ± 40.0	678.0 ± 40.0
Body weight gain, g		12.0 ± 1.0	35.0 ± 2.0^#^
Body energy, kJ	15.0 ± 0.6	15.5 ± 0.3	15.2 ± 0.7
Body lipids, g/100 g b.w.	21.6 ± 1.8	25.3 ± 1.6 *	26.7 ± 3.0 *
Body proteins, g/100 g b.w.	27.6 ± 1.8	23.6 ± 2.0 *	20.0 ± 2.0 *
Body water, g/100 g b.w.	58.5 ± 1.2	56.7 ± 0.9	55.5 ± 2.4
**Energy Balance**			
Food intake, g		868.0 ± 31.0	719.0 ± 29.0
Metabolizable energy intake, kJ		9524.0 ± 450.0	10,715.0 ± 864.0
Body energy gain, kJ		477.0 ± 40.0	598.0 ± 30.0 ^#^
Body lipid gain, kJ		1091.0 ± 100.0	1697 ± 100.0 ^#^
Body protein gain, kJ		−584.0 ± 30.0	−1068.0 ± 100.0 ^#^
Energy expenditure, kJ		9048.0 ± 400.0	10,117.0 ± 1000.0
Energetic efficiency, %		5.0 ± 0.2	5.5 ± 0.2

Values are the means ± SEM of eight rats. Body composition values: *p < 0.05 compared to initial values, ^#^*p* < 0.05 compared to low-fat diet (One-way ANOVA followed by Tukey post-test). Energy balance values: ^#^*p* < 0.05 compared to low-fat diet (two-tailed, unpaired, student’s t-test). b.w.= body weight.
